# Breast Conservative Surgery for Breast Cancer: Indian Surgeon’s Preferences and Factors Influencing Them

**DOI:** 10.1007/s13193-022-01601-y

**Published:** 2022-08-03

**Authors:** Rohini Dutta, Anshul Mahajan, Priti Patil, Geetu Bhandoria, Bhakti Sarang, Sargun Virk, Monty Khajanchi, Samarvir Jain, Lovenish Bains, Prashant Bhandarkar, Shamita Chatterjee, Nobhojit Roy, Anita Gadgil

**Affiliations:** 1 World Health Organisation Collaborating Centre for Research in Surgical Care Delivery in Low-Middle-Income Countries, Mumbai, India; 2 Christian Medical College and Hospital, Ludhiana, Punjab India; 3 Government Medical College Amritsar, Punjab, India; 4 Sri Guru Ram Das Institute of Health and Science, Amritsar, Punjab India; 5 Dayanand Medical College and Hospital, Ludhiana, Punjab India; 6 Maulana Azad Medical College, New Delhi, India; 7 Institute of Post-Graduate Medical Education & Research, Seth Sukhlal Karnani Memorial Hospital, Kolkata, India; 8 Department of Public Health Systems, Karolinska Institute, 171 77 Stockholm, Sweden; 9 The George Institute for Global Health, New Delhi, India

**Keywords:** Breast conservative surgery, Mastectomy, Breast cancer, Cancer surgery, India

## Abstract

**Background:**

It is well established that disease-free survival and overall survival after breast conservation surgery (BCS) followed by radiotherapy are equivalent to that after mastectomy. However, in Asian countries, the rate of BCS continues to remain low. The cause may be multifactorial including the patient’s choice, availability and accessibility of infrastructure, and surgeon’s choice. We aimed to elucidate the Indian surgeons’ perspective while choosing between BCS and mastectomy, in women oncologically eligible for BCS.

**Methods:**

We conducted a survey-based cross-sectional study in January–February 2021. Indian surgeons with general surgical or specialised oncosurgical training, who consented to participate were included in the study. Multinomial logistic regression was performed to assess the effect of study variables on offering mastectomy or BCS.

**Results:**

A total of 347 responses were included. The mean age of the participants was 43 ± 11 years. Sixty-three of the surgeons were in the 25–44 years age group with the majority (80%) being males. 66.4% of surgeons ‘almost always’ offered BCS to oncologically eligible patients. Surgeons who had undergone specialised training in oncosurgery or breast conservation surgery were 35 times more likely to offer BCS (*p* < 0.01). Surgeons working in hospitals with in-house radiation oncology facilities were 9 times more likely to offer BCS (*p* < 0.05). Surgeons’ years of practice, age, sex and hospital setting did not influence the surgery offered.

**Conclusion:**

Two-thirds of Indian surgeons preferred BCS over mastectomy. Lack of radiotherapy facilities and specialised surgical training were deterrents to offering BCS to eligible women.

**Supplementary Information:**

The online version contains supplementary material available at 10.1007/s13193-022-01601-y.

## Introduction

Breast cancer is the most common malignancy among women in India and around the world [1, 2]. In the year 2020, 178,361 new cases and 90,408 fatalities were attributed to breast cancer in India [2]. By 2025, breast cancer is projected to make up 15% of the nation’s total cancer burden [3, 4].Over decades, the standard surgical procedure to treat breast malignancy has been modified radical mastectomy (MRM). More recently, breast conservative surgery (BCS) with radiotherapy has gained widespread acceptance in the treatment of breast cancer all over the world. With advancements in surgical techniques, oncoplastic procedures and radiation therapy technology, survival rates after BCS followed by radiotherapy have been found to be equivalent to mastectomy, making breast cancer treatment less mutilating and cosmetically more acceptable for women [3, 4]. Despite this, the rate of BCS has been relatively low in Asian countries [5]. While the USA reported 64.5% of women with early-stage breast cancer undergoing BCS, 31% of breast carcinoma patients in Singapore underwent BCS [6, 7]. In India, only 11.3% of the patients who were offered BCS, underwent the procedure [8].

The strikingly low rates of BCS in low-and-middle-income countries (LMICs), including India, is because the majority of breast carcinoma cases are being detected at advanced stages as compared to high-income countries (HICs) [9–12]. Several infrastructure-related factors (availability and accessibility and affordability), shortage of surgeons with specialised BCS training, patient concerns about recurrence and radiation have also been documented to contribute to the preference of mastectomy over BCS [8, 13, 14]. Adjuvant radiotherapy facilities and multidisciplinary teams predominantly are concentrated in urban areas leading to resource mismatch [14]. Furthermore, 75% of cancer costs in India are out-of-pocket expenditures which contribute to delayed diagnosis and treatment [13]. Mandatory addition of adjuvant radiotherapy to BCS adds to this burden.

In Asia, although treatment options are discussed with patients, it is seen that the treatment decision is significantly based on the surgeon’s recommendations [15]. Even in tertiary care centres, the surgeon is documented as an independent determinant for mastectomy [16]. Therefore, understanding the factors that influence the treating surgeon’s choice of surgery is very important. There is a paucity of data on the factors that affect providers’ decisions in India. Thus, we conducted this study to elucidate the Indian surgeons’ choice of surgical treatment between BCS and mastectomy in oncologically eligible women [17].

## Methodology

### Study Design

We conducted a cross-sectional survey among surgeons practising in India, to assess their perception about choosing BCS and MRM in treating patients with breast cancer for 3 weeks between January and February 2021.

### Development and Pretesting

The survey was developed by a research consortium ‘IndSurg’ constituting practising surgeons and medical students, initiated by the WHO Collaborating Centre for Research in Surgical Care Delivery in LMICs, Mumbai, India. The survey was designed in the English language and questions were tested through a series of pilot surveys for unbiased intents and ambiguity. Data variables were chosen to include factors addressed in the literature and were objective, easily standardised and relevant to minimise missing data and maximise data quality. The survey was strictly anonymised and no identifying data were collected.

The survey had three sections (supplementary file). Variables such as details of surgical training, volume of breast cancer surgeries performed and area of practice listed in the survey were re-grouped during analysis as seen in the ‘Results’ section. The first section included questions about surgeons’ demographics, educational qualifications, area of expertise, training in oncosurgery and whether they practised in public (free service) or private (fee for service) hospitals [18, 19]. The second section enquired about the breast cancer work volumes, whether they offer BCS or MRM in women who are oncologically eligible to receive breast conservation surgery and their expertise and comfort in performing BCS [20]. The third section included reasons, if any, for choosing MRM over BCS, in patients who are eligible for breast conservation. This section included factors that would influence the surgeons’ decisions based on patient population, radiotherapy facilities available in the area of practice and their training and ability to perform BCS.

### Data Collection

This was an open (open access to anybody with the survey link) self-administered survey administered via ‘Google Forms’ that automatically captured the data. The survey link was circulated in various surgical groups on social media and email lists of professional associations and societies of surgeons and surgical oncologists. Survey link responses were active for 3 weeks between January 2021 and February 2021. Participation in the survey was voluntary after agreeing to consent to participate. No reminder emails and messages were sent to the participants. Surgeons who had either completed a masters degree in general surgery or underwent oncosurgical training during or following their masters in general surgery, and were practising in India were included in the study. Surgeons who did not consent to participate or are not performing any breast cancer surgery were excluded from the study. Participation in the survey was voluntary, without any incentive for participation.

### Statistical Analysis

Data were analysed using SPSS Version 24 and Microsoft Excel 2019. Descriptive statistics for overall study participants were presented as absolute numbers and percentages of the group. Multinomial logistic regression was performed to assess the effect of study variables on offering BCS to an eligible patient. A *p*-value of less than 0.05 was considered statistically significant.

## Results

A total of 351 responses were received at the end of the study period. Four surgeons did not perform breast surgery as a part of their clinical practice, so were excluded from further analyses yielding a final sample of 347 respondents. Since the survey was circulated through multiple platforms, we were unable to calculate a response rate.

### Participant Characteristics

The mean age of the study participants was 43 ± 11 years with 63.1% of the participants in the 25–44 years age group. The majority of participants (80.5%) was males. 59.8% had received specialised oncosurgical training. A quarter (25.6%) of the study participants reported that they were practising breast surgery for the last 20 years or more and 137 (42.2%) surgeons mentioned that they perform more than ten breast cancer surgeries in a month as a lead surgeon (Table 1). Of the 207 participants with oncosurgical training, 191 had done a fellowship or masters course in oncosurgery and 16 had BCS training as a part of their masters/residency training in general surgery.

**Table 1:** Participant characteristics

Variable	*N* = 347	%
Age, years: mean ± SD (range)	43 ± 11	(25–83)
Age group (years)		
25–34	90	25.9%
35–44	129	37.2%
45–54	71	20.5%
55–64	41	11.8%
≥ 65	16	4.6%
Sex of participant		
Male	276	80.5%
Female	67	19.5%
Details of surgical training		
Surgeons with oncosurgery training	207	59.8%
Masters in general surgery	139	40.2%
Setting of practice		
Private	194	56.1%
Public	152	43.9%
Level of institution		
Tertiary hospital	303	88.1%
Secondary hospital	41	11.9%
Duration of surgical practice (years)		
< 10	150	43.5%
10–20	106	30.7%
> 20	89	25.8%
The volume of breast cancer surgeries in your practice per month as a lead surgeon		
< 10 surgeries	188	57.8%
≥ 10 surgeries	137	42.2%
Distance of radiation oncology facility from your institute/place of practice		
Same institute	209	60.8%
Not in the same institute but in the same city	128	36.8%
How likely are you to offer BCS to an eligible woman?		
Almost always	229	66.4%
Occasionally	91	26.4%
Almost never	25	7.2%

### Surgeons’ Choice Between BCS and Mastectomy

Two-thirds (66.4%) of the surgeons almost always offered BCS to every eligible woman (Table 1). Various reasons for not offering BCS were expressed by the surgeons, even if the woman was oncologically eligible for BCS (Table 2).

**Table 2:** Reason for choosing mastectomy over BCS

Choosing MRM over BCS reason	*N* = 261	%
My patients may not follow up for radiation after surgery	109	31.4%
My patients cannot afford BCS and adjuvant radiation	61	17.6%
Believe mastectomy is an oncologically safer option	42	12.1%
No radiotherapy facilities where I practice	24	6.9%
I lack the training or expertise to perform BCS	20	5.7%
No mammography facility where I practice	5	1.4%

*Numbers and percentages may not add up due to multiple reasons given by many surgeons

### Factors Associated with Surgeons’ Decision on Treatment

Multinomial logistic regression was used to predict the independent factors that would decide the type of surgery from the surgeon’s perspective (Table 3). The relationship between dependent variables (offering BCS to an eligible woman) was probed by considering the ‘almost never offering BCS’ as a reference category. Surgeons with specialised oncosurgical training had significantly higher chances of offering BCS than general surgeons (occasionally offering BCS OR 8.27, *p* = 0.059 and almost always offering BCS OR 35.24, *p* = 0.001). There was no association of the surgeons’ demographic details (age, sex) and years of practice on their preference of surgical procedure. Surgeons demonstrated seven and nine times higher odds of ‘occasionally’ and ‘almost always’ offering BCS respectively if the radiation oncology facility was available in the same institute (OR = 7.488, *p* = 0.044, OR = 9.655, *p* = 0.025).

**Table 3:** Multinomial logistic regression—how likely are surgeons to offer BCS to an eligible woman

Narration	Almost never (referent category)	Occasional			Almost always		
	*n*	*n*	Odds ratio (95% CI)	*p*-value	*n*	Odds ratio (95% CI)	*p*-value
Age group (years) (*N* = 345)							
25–34	9	26	0.314 (0.006–15.232)	0.559	54	0.211 (0.004–10.605)	0.436
35–44	5	34	0.5 (0.013–19.143)	0.709	89	0.218 (0.005–8.694)	0.418
45–54	5	19	0.539 (0.029–9.903)	0.677	47	0.303 (0.016–5.746)	0.427
55–64	4	8	0.436 (0.03–6.332)	0.543	29	0.641 (0.045–9.134)	0.743
> 65	2	4	Referent category		10	Referent category	
Sex of participant (*N* = 341)							
Female	5	17	1.558 (0.351–6.917)	0.56	45	1.615 (0.36–7.243)	0.531
Male	20	72	Referent category		182	Referent category	
Details of surgical training (*N* = 344)							
Surgeons with oncosurgery training	1	32	8.273 (0.927–73.845)	0.059	173	35.236 (4.059–305.862)	0.001
Masters in general surgery	24	59	Referent category		55	Referent category	
Practice centre (*N* = 344)							
Public	14	53	1.033 (0.328–3.252)	0.955	83	0.49 (0.153–1.564)	0.228
Private	11	38	Referent category		145	Referent category	
Centre level (*N* = 342)							
Tertiary hospital	19	80	0.814 (0.177–3.739)	0.791	202	0.993 (0.209–4.724)	0.993
Secondary hospital	6	11	Referent category		24	Referent category	
Duration of practising surgery (*N* = 343)							
< 10 years	10	43	3.69 (0.212–64.352)	0.371	95	3.213 (0.182–56.684)	0.425
10–20 years	6	26	1.039 (0.134–8.035)	0.971	74	0.873 (0.111–6.866)	0.897
> 20 years	8	21	Referent category		60	Referent category	
The volume of breast cancer surgeries performed in a month as a lead surgeon (*N* = 323)							
< 10 surgeries	19	66	0.961 (0.174–5.321)	0.964	103	0.443 (0.083–2.366)	0.34
≥ 10 surgeries	2	21	Referent category		112	Referent category	
Distance to the radiation oncology facility from institute/place of practice (*N* = 337)							
Same institute	5	49	7.488 (1.059–52.936)	0.044	153	9.655 (1.325–70.374)	0.025
Not in the same institute but in same city < 2 h	14	31	1.637 (0.33–8.135)	0.547	64	1.724 (0.329–9.043)	0.52
In same city > 2 h	5	7	Referent category		9	Referent category	

*Two participants have not responded to the offering of BCS question, those were not considered in the multinomial logistic regression analysis

## Discussion

Our study explored the Indian surgeons’ perspective while offering BCS to oncologically eligible women. It was seen that 66.4% of surgeons almost always offered BCS to oncologically eligible women. Specialised surgical training and distance of the radiation oncology facility from the surgeon’s institute were the independent determinants for choosing BCS over mastectomy.

Our study highlighted that surgeons with oncosurgical training almost always offered BCS to oncologically eligible patients. This was similar to studies from India (*p* < 0.01) and China (*p* = 0.003) which found that surgeons with super speciality surgical training were performing BCS more frequently compared to general surgeons and were independent predictors of BCS [8, 21]. In contrast, a study from the USA reports that surgical oncologists were more likely to perform mastectomy over BCS. However, it was also reported that this could be a reflection of the fact that they treat more complicated patients [22].

The perceived inability of patients to follow up for adjuvant radiotherapy was cited as one of the most common reasons for not offering BCS in our study. Surgeons were nine times more likely to almost always offer BCS to patients if radiotherapy facilities were available in the same institute compared to if the radiation facility was within the same city more than 2 h away (*p* = 0.01). A similar trend was seen in a study from the USA where the likelihood of BCS increased when a radiation facility was available in the same hospital [23]. The study also demonstrated that women who underwent BCS were less compliant to the use of adjuvant radiation therapy if they lived greater distances (≥ 40 miles) from a centre with a radiation facility. This is also in concordance with a study from Iran where the non-availability of adjuvant radiotherapy facilities was cited as one of the reasons for surgeons not recommending BCS to their patients [24]. This highlights that access to adjuvant radiotherapy services is a major determinant of BCS and warrants prioritisation of investment in radiotherapy facilities to increase its access and affordability.

In North America, female surgeons were found more likely to offer BCS, as they were supposedly able to alleviate patients’ concerns regarding BCS better in comparison to their male counterparts [25]. Female surgeons were twice more likely to offer BCS than males as shown in an analysis by Hershman et al. in patients with early-stage breast cancer [26]. However, our study did not show any variation in the outlook towards BCS according to surgeons’ sex or age. This was similar to another study from India done by Bothra et al. [19]. We did not find a statistically significant association between surgeons’ years of experience with their decision to offer BCS. Conversely, Bothra et al. highlighted that Indian surgeons during the early part of their career (age group of 20–30 years) preferred to perform mastectomy over BCS due to the perceived fear of tarnishing reputation in case of failure of treatment. Senior surgeons (age > 50 years) preferred to perform mastectomy due to their lack of training in BCS [19]. Arnaud et al. also found that patients treated by older surgeons underwent mastectomy more often [27].

Our study has a large sample size and a good representation of participating surgeons across age and years in practice. However, it has several limitations. A majority of participants (81%) included in our study practised in tertiary care centres, whereas the majority of the breast cancer surgeries is performed by the general surgeons at secondary-level hospitals or private hospitals, in India. Similarly, rural areas where 70% of India’s population resides may not have surgeons trained in BCS or access to radiotherapy. This survey thus may underrepresent the surgeons of rural areas, private hospitals and smaller setups where significant volumes of breast surgeries are performed [14]. It provides insight into why surgeons may hesitate to offer BCS to eligible women, but does not take into account violations from standard practice, such as the number of times surgeons did not offer BCS to eligible patients, as our survey focussed primarily on the surgeons’ perspective and relies on their honesty in answering. Additionally, the knowledge gap of surgeons was ascertained based on their level of education and training as per the participants’ responses. However, a nuanced approach to look into factors determining these reasons should be explored in future studies.

A surgeons’ intent to impart appropriate quality of treatment and improved quality of life for the patient is essential for performing BCS in an eligible patient [8]. Therefore, general surgeons with less exposure to BCS training need to be trained in performing BCS and in counselling patients. This can occur through the inculcation of training in BCS as a part of general surgical training. Higher fellowships or specialised oncosurgical training after completion of masters in general surgery will always remain an option for a section of surgeons. Previous studies from Hong Kong and Malaysia have found that most patients follow their surgeon’s recommendations in deciding between mastectomy and BCS [28, 29]. This makes it essential for patients with breast cancer to be informed of all their treatment options by the surgeons, to make an informed surgical decision. This can be supported through the implementation of shared decision-making tools, which have been shown to increase the rate of BCS [30, 31].

The data from our study shows that the majority of surgeons offers BCS to eligible patients. However, it is observed that a significant number of women who could be candidates for BCS still decide to undergo mastectomy [14]. Hence, larger-scale studies analysing and documenting the effect of multiple factors must be conducted to understand surgical care for breast cancer in India. Future work should focus on identifying intricate barriers pertaining to access and availability of radiation treatments that may influence patients’ choice of BCS [8, 24].

## Conclusion

We found that two-thirds of Indian surgeons prefers BCS over mastectomy. This is consistent with global trends. Lack of surgeons trained in performing BCS was a common deterrent to offering BCS. This requires scaling of training opportunities in BCS for general surgeons. Availability of a radiation facility in the same institute as the treating surgeon was the top determinant in surgeons offering BCS to oncologically eligible patients. Investment in radiotherapy facilities to increase access and affordability must be prioritised to further increase BCS rates in India.

## Supplementary Information

Below is the link to the electronic supplementary material.

Supplementary file1 (PDF 164 KB)

## Data Availability

The dataset generated and analysed during the current study are available from the corresponding author on reasonable request.
