# Supplementary material for: Breast Conservative Surgery for Breast Cancer: Indian Surgeon’s Preferences and Factors Influencing Them
Source: Indian J Surg Oncol. 2022 Aug 3;14(1):11–7. doi: 10.1007/s13193-022-01601-y (PMC9986359; doi:10.1007/s13193-022-01601-y)
Supplement: Supplementary file 1 — Supplementary file1 (PDF 164 KB) [file 13193_2022_1601_MOESM1_ESM.pdf]

Which Surgery Do Indian Surgeons Prefer, Mastectomy or Breast Conservation?

It is observed that mastectomy (MRM) is chosen over breast-conserving surgery (BCS) in case of 'early-stage' breast cancer, in India. We aim to analyze the surgeons' preferences and reasons for offering a particular type of surgery. (Breast-conservation v/s mastectomy). This nationwide survey will take 3 to 4 minutes to fill in and help us understand the factors behind these choices. Many thanks for choosing to answer this survey. You are consenting to use of your responses in the research to determine factors in choosing BCS vs MRM, by answering the survey. The results of this study will be communicated at respective society conferences, meetings, social media and as publication in specialists journals . All responses will remain anonymized and no personal data like e-mail addresses etc. will be stored at the end of the project. We thank you for your valuable time.
Please do not answer this survey if breast surgery is not part of your clinical practice.

*Required

1. Consent to participate: By clicking this button I choose to participant in survey to identify preference of surgeons in MRM and BCS. I agree that survey will use anonymous responses and I voluntary choose to participate. *

Mark only one oval.

- Yes *Skip to question 2*
- No

Section 2

2. Whether breast surgery is a part of your clinical practice?

Mark only one oval.

- Yes *Skip to question 3*
- No

Skip to question 3

Responder details

This section has questions related to your age, educational qualifications, area of expertise, years of experience and the geographical area of practice

3. 1. What is your area of work / expertise? *

Mark only one oval.

- ( ) Family physician/ General physician
- ( ) General surgeon
- ( ) Oncosurgeon
- ( ) Specialized breast surgeon/Onco-plastic surgeon
- ( ) Surgical trainee/resident
- ( ) Plastic or reconstructive surgeon

4. 2. What is your age ? mention age in years *

____

5. 3. Sex of the responder

Mark only one oval.

- ( ) Female
- ( ) Male
- ( ) Prefer not to say

6. 4. Details of Surgical training *

Tick all that apply.

- [ ] MBBS
- [ ] MS/MD/DNB Surgery
- [ ] MCh/ DM or any other masters degree in oncology
- [ ] Fellowship/Short duration courses in oncology
- [ ] Received BCS training in unit where BCS was offered commonly

7. 5. Where do you practice? *

Mark only one oval.

- Reginal cancer center
- Private/Corporate tertiary care hospital
- Public/Government tertiary care hospital
- Secondary/District hospital
- Private nursing home/Clinic
- Other: ______________________

8. 6. Area of your practice as a care giver *

Tick all that apply.

- [ ] Rural
- [ ] Semiurban
- [ ] Urban

Skip to question 9

Choosing Mastectomy vs. Breast Conservation Surgery

This section enquires about the factors influencing surgeons' decisions to choose between mastectomy and BCS

9. 7. How long have you been practicing surgery?

Mark only one oval.

- <10years
- 10-20 years
- .20 years

10. 8. Do you perform cancer surgery? *

Mark only one oval.

- Yes
- No

11. 9. What is the volume of breast cancer surgeries in your practice per month as a lead surgeon?

Mark only one oval.

- <10
- 10-20
- >20
- Not applicable

12. 10. How likely are you to offer BCS to an eligible woman?

Mark only one oval.

- Almost always
- Occasionally
- Almost never

13. 12. Do you offer oncoplastic or reconstructive procedures for breast cancer, either yourself or with the help of a colleague?

Mark only one oval.

- Almost always
- Occasionally
- Almost never

14. 13. Do you offer conservative axillary management like sentinel node or axillary sampling, yourself or with the help of a colleague?

Mark only one oval.

- Almost Always
- Occasionally
- Almost never

15. 14. How far is the radiation oncology facility from your institute/place of practice?

Mark only one oval.

- Available in the same institution
- Not available at my hospital but available in the same town/city, under 2 hours distance
- Available in same town/city, beyond 2 hours distance
- Other: ______________________

16. 15. Why would you choose MRM over BCS even if the woman is oncologically eligible for BCS?

Tick all that apply.

- [ ] I Lack training or expertise to perform BCS
- [ ] Believe MRM is oncologically safer option
- [ ] No mammography facility where I practice
- [ ] No radiotherapy facilities where I practice
- [ ] My patients cannot afford BCS and adjuvant radiation
- [ ] My patients may not follow up for radiation and after surgery
- [ ] Not applicable , I am offering BCS to every eligible patient every

We thank you for your valuable time and efforts

This content is neither created nor endorsed by Google.

Google Forms
